# A Novel Antiviral Therapeutic Platform: Anchoring IFN-β to the Surface of Infectious Virions Equips Interferon-Evasive Virions with Potent Antiviral Activity

**DOI:** 10.3390/v17050697

**Published:** 2025-05-13

**Authors:** Hoda H. Jabbour, Alexander G. Bastian, Kayla B. DeOca, Mark D. Mannie

**Affiliations:** Department of Microbiology and Immunology, Brody School of Medicine, East Carolina University, Greenville, NC 27834, USAkayla.b.deoca@gmail.com (K.B.D.)

**Keywords:** COVID-19, SARS-CoV-2, NL63, ACE2, IFN-β, platform technology, fusion protein, type I interferon, flow cytometry, drug discovery, antivirals, emerging/re-emerging RNA viral pathogens, immunomodulators

## Abstract

The COVID-19 pandemic highlighted the need for new therapeutic strategies to counter emerging pathogenic viruses. Herein, we introduce a novel fusion protein platform that enables antiviral targeting of distinct viral species based on host receptor specificity. Proof-of-concept studies focused on the human coronavirus NL63, which shares specificity for the ACE2 host receptor with the pandemic SARS-CoV and SARS-CoV-2 species. This antiviral fusion protein combines IFN-β with the soluble extracellular domain of ACE2 (IFNβ-ACE2). Both domains retained predicted bioactivities in that the IFN-β domain exhibited potent antiproliferative activity and the ACE2 domain exhibited full binding to the transmembrane SARS-CoV-2 Spike protein. In virus-washed (virus-targeted) and non-washed in vitro infection systems, we showed that the pool of IFNβ-ACE2 targeted to the virion surface had superior antiviral activity against NL63 compared to soluble ACE2, IFN-β, or the unlinked combination of ACE2 and IFN-β. The pool of IFNβ-ACE2 on the virion surface exhibited robust antiviral efficacy based on the preemptive targeting of antiviral IFN-β activity to the proximal site of viral infection. In conclusion, virus-targeted IFN-β places interferon optimally and antecedent to viral infection to constitute a new antiviral strategy.

## 1. Introduction

The severe acute respiratory syndrome coronavirus 2 (SARS-CoV-2) pandemic is driven by the repeated emergence of new pathogenic variants that continually diversify under evolutionary pressure from the global pool of anti-SARS-CoV-2 immunity [1,2,3,4,5,6,7,8,9]. The evolutionary drive on pathogenic variants is shaped by the lack of sterilizing immunity in infected and/or vaccinated individuals and particularly in immunocompromised individuals who harbor replicating virus for prolonged periods. Rapid evolution of SARS-CoV-2 is compounded by the inherent mutability of the viral genome which is driven via the accumulation of point mutations and genetic recombination with other circulating variants [10,11,12]. Both types of mutations compromise the affinity and neutralization capacity of pre-existing anti-Spike antibodies in the human population, thereby generating immune escape variants. Notably, however, all emergent pathogenic variants continue to share specificity for the angiotensin-converting enzyme 2 (ACE2) host receptor, thereby providing a means to target all SARS-CoV-2 variants and all other viral species sharing the ACE2 host receptor specificity.

To exploit the common ACE2 host receptor specificity, we developed a novel antiviral platform comprising the antiviral effector cytokine interferon-beta (IFN-β) fused to the soluble extracellular domain of ACE2 (sACE2). This IFNβ-ACE2 fusion protein is predicted to bind human coronaviruses (HCoV) NL63, SARS-CoV, and SARS-CoV-2 [13,14,15,16,17,18] via the virion surface Spike glycoprotein. The rationale is that the physical coupling of the IFN-β and sACE2 domains will confer synergistic activities not realized by either domain acting alone, based on the following postulates: (a) The sACE2 domain is predicted to anchor IFN-β to the virion surface and thereby coat the virion with a surface array of IFN-β. (b) Upon interaction of a partially neutralized virion with a host target cell, the IFN-β domain anchored to the surface of the virion is predicted to trigger interferon-α/β receptor (IFNAR) signaling before host cell infection, such that the virion will only infect host cells with previously upregulated type I interferon (IFN-I) antiviral defenses. (c) Induction of IFN-I defenses prior to infection is predicted to abrogate subsequent viral replication, thereby blocking amplification of viral cytopathic effects and cytokine-storm immunopathogenesis. Given that HCoV NL63, SARS-CoV, and SARS-CoV-2 robustly block IFN-β production [19,20,21,22,23,24,25,26,27], delivery of exogenous IFN-β via an IFNβ-ACE2 targeting mechanism is predicted to obviate a primary immune evasion tactic of coronaviruses, thereby blocking viral infection and systemic dissemination. Clinical application of IFNβ-ACE2 via nebulized aerosols directly to the pulmonary system would be of potential benefit during infection with ACE2-targeting viruses in patients who are asymptomatic, pre-symptomatic, or presenting with mild to severe disease including ARDS or cytokine-release/vascular-leak immunopathology.

Although engineering a virion surface-targeted IFN-β array is an attractive antiviral strategy, the concept requires experimental validation. Potential pitfalls include the following: (a) The IFN-β domain arrayed on the virion surface may not have the appropriate molecular orientation to efficiently engage IFNAR on the host cell to initiate antiviral signaling. (b) The concentration of IFN-β array at the virion surface may be insufficient to trigger a protective antiviral response. (c) The virion-surface IFN-β array may not provide an adequate lead time to establish antiviral innate immunity before viral infection. (d) The IFN-β domain may augment viral infectivity by bridging virus and host cell membranes. To address these potential concerns, in vitro infection studies were used to test the conceptual basis of the IFNβ-ACE2 modality. These experiments revealed that IFNβ-ACE2 had enhanced antiviral activity against NL63 compared to sACE2, IFN-β, and the unlinked combination of ACE2 and IFN-β. Additionally, in vitro targeting studies were performed in which NL63 virions were incubated with IFNβ-ACE2 and then washed to remove unbound proteins to test the antiviral activity of IFNβ-ACE2 physically linked to the viral surface. These ‘virus-washed’ experiments revealed that virion surface-bound IFNβ-ACE2 had potent antiviral activities that were superior compared to either or both sACE2 and/or IFN-β. Additionally, there was no evidence that IFNβ-ACE2 enhanced infection by bridging the virus and host cell membranes. The main conclusion is that the IFNβ-ACE2 array on the virion surface has potent antiviral activity due to the preemptive targeting of antiviral IFN-β activity to the exact site and time of imminent viral infection. Thus, virus surface-targeted IFN-β provides a conceptual basis for a new platform technology and a new category of antiviral therapy.

## 2. Materials and Methods

### 2.1. Protein, Virus, and Cells

Human IFN-β (300-02BC) was purchased from Peprotech (Cranbury, NJ, USA) and is referenced as ‘IFN-β (Peprotech)’. *Cercopithecus aethiops* kidney epithelial cells expressing transmembrane protease, serine 2 and human angiotensin-converting enzyme 2 (Vero E6-TMPRSS2-T2A-ACE2 cells) (NR-54970), *Homo sapiens* lung carcinoma (A549) cells (NR-52268), human coronavirus NL63 (NR-470), and human coronavirus 229E (NR-52726) were obtained from BEI Resources (Manassas, VA, USA). LLC-MK2 cells (CCL-7) were purchased from ATCC. Vero E6-TMPRSS2-T2A-ACE2, A549, and LLC-MK2 cells were cultured in Dulbecco’s modified Eagle’s medium (DMEM) supplemented with 10% fetal bovine serum (FBS). Human embryonic kidney 293-F (HEK) cells (R79007, Gibco, Waltham, MA, USA) were cultured in FreeStyle 293 Expression Medium (12338018, Gibco). Genes encoding full-length SARS-CoV-2 Spike (D614G) (VG40589-UT, Sino-Biological, Beijing, China) and full-length CD25 [28] were cloned into a pIRES AcGFP1 expression vector (632435, TaKaRa Bio, San Jose, CA, USA), and these vectors were used to transfect HEK cells to derive stable lines (HEK-Spike and HEK-control, respectively) that expressed the respective transmembrane proteins. Because stably transfected cells expressed GFP, flow cytometric analyses focused on HEK cells in the viable GFP^+^ gate. TF-1 cells (CRL-2003, ATCC) were cultured in RPMI supplemented with 5% FBS and 1 nM GM-CSF. The NL63 and 229E viruses were propagated on LLC-MK2 and A549 cells, respectively, and were concentrated at 4 °C by either ultracentrifugation at 48,000× *g* for 5 h or ultrafiltration on YM100 membranes. Viruses were then aliquoted and frozen at −80 °C.

### 2.2. Tissue Culture Infection Dose 50 (TCID_50_)

Infectious titers of NL63 and 229E were quantified in 96-well plate plaque assays in Vero E6-TMPRSS2-T2A-ACE2 cells and MRC-5 cultures, respectively. Cells were seeded at 30,000 cells/well and incubated at 33 °C. After 24 h, the cells were inoculated with a half-log serial dilution of the virus stock (8 replicates per dilution) and incubated at 33 °C. At 5 days post-infection, the supernatant was discarded, and the cell monolayer was washed with PBS and stained with crystal violet. After 15 min of staining, the crystal violet was discarded, the number of wells with plaques was counted for each dilution, and the TCID_50_ was calculated according to the Reed–Münch method [29].

### 2.3. Generation and Purification of Recombinant Proteins

We used stable mammalian expression systems for recombinant proteins. In addition to IFN-β (Peprotech), we expressed a recombinant IFN-β control protein (referenced as recombinant IFN-β) that comprised the human IFN-β sequence (P01574), except that a non-native alanine residue was added as the second N-terminal amino acid to encode an optimal Kozak translation-initiation site (GCCGCCACC-ATG-GCC-). To control for the enterokinase linker and poly-histidine sequence in IFNβ-ACE2, the C-terminus of recombinant IFN-β included an enterokinase linker cleavage site (GDDDDKG) and a C-terminal histidine purification tag (TRTSTHHHHHHHH). Thus, IFNβ-ACE2 and recombinant IFN-β represented a controlled comparison because both shared similar linker sequences. The IFNβ-ACE2 (P01574 and Q9BYF1, respectively) included an N-terminal Rat Albumin Signal Sequence (MAKWVTFLLLLFISGSAFS), the secreted human N-terminal IFN-β sequence (MSYN….GYLRN), an enterokinase cleavage site (GDDDDKG), a 9-histidine purification tag, and the human C-terminal sACE2 metallopeptidase domain (18-611) (GSTIE….STDWS). The sACE2(18-611) and sACE2(18-740) control proteins were composed of the human ACE2 sequence (Q9BYF1). These sequences included an N-terminal Rat Albumin Signal Sequence (MAKWVTFLLLLFISGSAFS), a 9-histidine purification tag, the respective sACE2 18-611 or 18-740 sequence, a linker (AKGGGSEGGGSEGGGSG), and a C-terminal GFP sequence (P42212). These constructs were cloned into a pIRES AcGFP1 expression vector (632435, TaKaRa Bio). HEK cells were stably transfected with these vectors and grown in FreeStyle 293 Expression Medium (Gibco). Expression supernatants were concentrated and buffer exchanged into 50 mM NaH_2_PO_4_ (pH 8.0) on YM10 ultrafiltration membranes and directly applied to Ni-NTA Agarose columns (Qiagen, Chatsworth, CA, USA) followed by extensive washing of the resin (50 mM NaH_2_PO_4_ with 0, 20, 40, or 60 mM imidazole, pH 8.0). IFN-β, sACE2(18-611), sACE2(18-740), and IFNβ-ACE2 were eluted with 50 mM NaH_2_PO_4_ with 250 mM imidazole (pH 8.0), concentrated in Amicon Ultra-15 centrifugal filter devices (EMD Millipore, Billerica, MA, USA), and dialyzed in Slide-A-Lyzer dialysis cassettes G2 (87735, ThermoFisher Scientific, Waltham, MA, USA). The protein was quantified by absorbance at 280 nm, and the purity was assessed by sodium dodecyl sulfate–polyacrylamide gel electrophoresis (SDS-PAGE).

### 2.4. Validation of the IFN-β Domain of IFNβ-ACE2: IFN-β Antiproliferation Assay

The IFN-β bioactivities of recombinant proteins were measured in vitro by inhibition of GM-CSF-dependent proliferation of TF-1 cells. TF-1 cells were incubated with 1 nM GM-CSF and IFNβ-ACE2, IFN-β (Peprotech), recombinant IFN-β, or sACE2(18-611) in complete RPMI for 3 days at 37 °C. The culture was pulsed with 1 μCi [^3^H]thymidine after 48 h of the 72 h culture. Cells were harvested onto filters by use of a Tomtec Mach III harvester (Hamden, CT, USA). Proliferation was measured via [^3^H]thymidine incorporation into DNA using a Perkin Elmer MicroBeta2 liquid scintillation counter.

### 2.5. Validation of the sACE2 Domain of IFNβ-ACE2: sACE2 Binding to HEK-Spike

The sACE2 domain was assessed by binding to HEK-Spike cells. HEK-Spike or HEK-control cells (100,000 cells/tube) were incubated with either sACE2(18-611) or IFNβ-ACE2 at 4 °C. After a 1 h incubation, cells were washed twice with PBS with 2% FBS and blocked with Fc Receptor Binding Inhibitor Polyclonal Antibody (14-9161-73, Invitrogen, Carlsbad, CA, USA) for 1 h at 4 °C. Following the blocking step, cells were washed twice with PBS with 2% FBS and stained with Alexa Fluor 647 (AF647)-conjugated rat anti-human ACE2 (375803, Biolegend, San Diego, CA, USA) for 1 h at 4 °C. Cells were then washed with PBS with 2% FBS and analyzed for surface-bound anti-ACE2 mAbs on a Cytek Aurora Spectral Cytometer (Fremont, CA, USA) with unmixing via unstained and single-stained control samples followed by analysis with De Novo Software FCS Express 7 (Glendale, CA, USA). The percentage of ACE2-positive cells was gated as the number of viable, single, live, GFP^+^ (stably transfected), and ACE2^+^ cells divided by the total number of cells in the GFP^+^ gate. Mean fluorescence intensity (MFI) of anti-ACE2 fluorescence was gated on all cells in the viable, single, live, and GFP^+^ gate.

### 2.6. Inhibition Assays of HCoV NL63 and 229E Infection In Vitro

The antiviral activity of IFNβ-ACE2 was tested using HCoV NL63 and 229E infection assays on Vero E6-TMPRSS2-T2A-ACE2 and A549 cells, respectively. Cells were seeded into a 96-well plate at 30,000 cells/well and grown for 24 h at 33 °C. NL63 and 229E were used at a multiplicity of infection (MOI) of 0.1. NL63 and 229E were incubated with either IFNβ-ACE2, sACE2(18-611), sACE2(18-740), IFN-β (Peprotech), recombinant IFN-β, or the unlinked combination of IFN-β and sACE2(18-611) for 1 h at 4 °C. For the ‘no wash’ system, the virus and protein were then added directly to the cells in that both virus-bound and non-bound protein were carried into the infection culture. For the ‘virus-washed’ system, the virus and protein mixtures were subjected to five ultrafiltration washing steps (300 kDa MWCO Nanosep centrifugal filters, OD300C34, Cytiva, Marlborough, MA, USA) in PBS with 0.5% poloxamer (sheer protectant) (pH 7.5). This virus-washing protocol retained virus and virus-bound protein in the retentate but eliminated unbound protein in the filtrate. Five washes eliminated unbound protein while maintaining viral infectivity, based on calculations as well as flow cytometry analyses of infectivity. The ‘washed’ virus preparations in the retentate were then added to the cells. For both non-washed and virus-washed protocols, virus and host cells were cultured for 2 days at 33 °C. After 2 days, cells were detached using TrypLE Express Enzyme (12563029, Gibco), transferred to a V-bottom 96-well plate, and blocked with Fc Receptor Binding Inhibitor Polyclonal Antibody (14-9161-73, Invitrogen) for 1 h at 4 °C. Control (non-infected) cells and virally infected cells were stained with a 1:1000 dilution of LIVE/DEAD Fixable Blue Dead Cell Stain Kit (L23105, Invitrogen) for 1 h at 4 °C. After washing with PBS with 5% FBS, Vero E6-TMPRSS2-T2A-ACE2 cells were surface-stained with 1:1600 dilution of PE-conjugated mouse anti-human TMPRSS2 (378403, Biolegend) and 1:100 dilution of FITC-conjugated mouse anti-human ACE2 (10108-MM36-F, Sino Biological) for 1 h at 4 °C and then were washed twice with PBS with 5% FBS. For intracellular staining, Vero E6-TMPRSS2-T2A-ACE2 and A549 cells were fixed and permeabilized using eBioscience Foxp3/Transcription Factor Staining Buffer Set (00-5523-00, Invitrogen) by incubating cells in the Fixation/Permeabilization buffer for 30 min at room temperature. Cells were then washed twice with the Permeabilization buffer and blocked with 10% rabbit serum (16120-099, Gibco) for 1 h at 4 °C. Antibodies for intracellular staining of nucleocapsid protein were previously prepared by conjugating rabbit anti-NL63 nucleocapsid antibody (40641-T62, Sino Biological) or rabbit anti-229E nucleocapsid antibody (40640-T62, Sino Biological) with AF647 by use of the AF647 Conjugation Kit (Fast)—Lightning-Link (ab269823, Abcam). Intracellular staining of cells infected with HCoV NL63 and 229E was performed by incubating cells with 1:1600 dilution of AF647-conjugated rabbit anti-NL63 nucleocapsid antibody or 1:1600 dilution of AF647-conjugated rabbit anti-229E nucleocapsid antibody, respectively, for 1 h at 4 °C. After washing once with permeabilization buffer and once with PBS with 2% FBS, cells were resuspended in PBS with 2% FBS and analyzed on a Cytek Aurora Spectral Cytometer (Fremont, CA, USA) with unmixing via unstained and single-stained control samples followed by analysis with De Novo Software FCS Express 7 (Glendale, CA, USA). Cells were gated on viable, single, and live cells before gating on FITC^+^, PE^+^, and AF647^+^ gates. MFI of PE and FITC was gated on all viable, single, and live cells.

### 2.7. Statistical Analyses, Data Presentation, and Experimental Reproducibility

Comparisons with one independent variable were assessed via one-way analysis of variance (ANOVA) with the Dunnett multiple comparisons test. Comparisons with two independent variables were assessed via two-way ANOVA with Tukey’s multiple comparisons test. A *p*-value < 0.05 was considered significant. Significance was indicated as follows: (* or ° *p* < 0.05, ** or °° *p* < 0.01, *** or °°° *p* < 0.001, and **** or °°°° *p* < 0.0001). Each data point represents the mean value, and error bars represent the standard deviation (SD). Experiments shown in the figures are representative of three independent experiments.

## 3. Results

**Figure 1:**
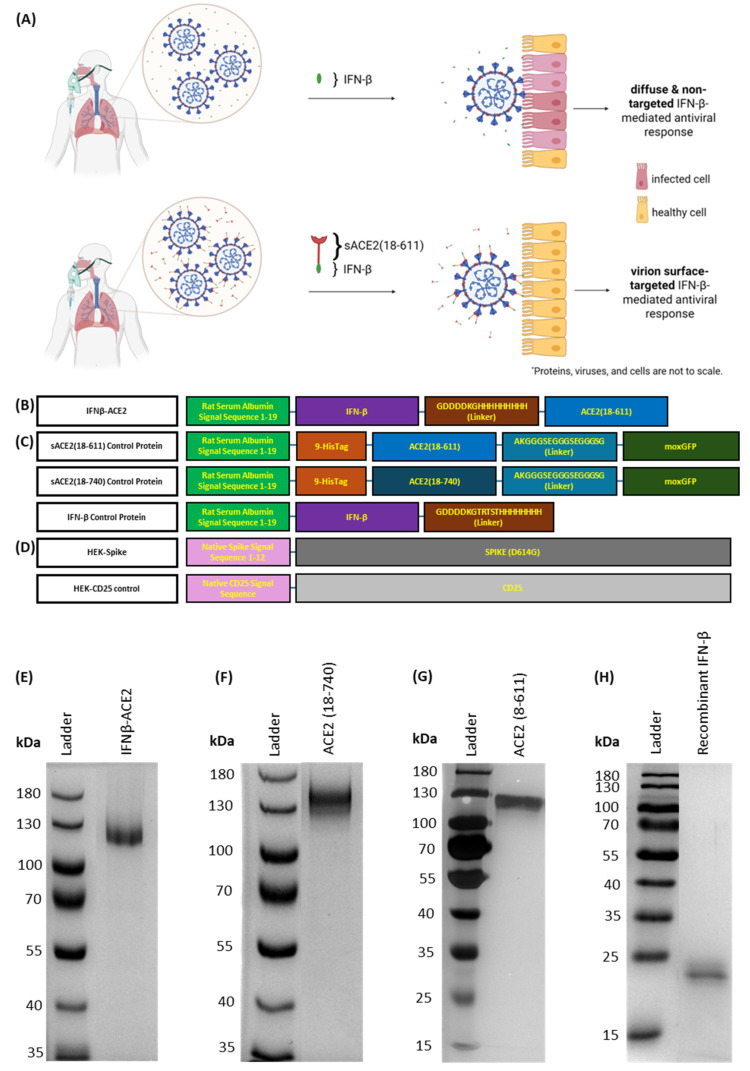
**Postulated mechanism underlying the IFNβ-ACE2 fusion protein.** (**A**) A SARS-CoV-2- or NL63-infected individual is given the IFNβ-ACE2 via nebulization to the lungs. The sACE2 domain is postulated to bind the Spike protein and coat the virion with a surface array of IFN-β. Based on this strategy, the IFN-β domain will drive IFN-β signaling pathways and antiviral activity in the target cell before viral entry. IFNβ-ACE2 is postulated to provide a more concentrated and targeted approach to delivering IFN-β to the exact site and time of imminent viral infection [30]. (**B**) Schematic diagram of the fusion protein construct consisting of an IFN-β domain and a sACE2(18-611) domain. (**C**) Schematic diagrams of the soluble control proteins: sACE2(18-611), sACE2(18-740), and IFN-β. (**D**) Schematic diagrams of the transmembrane proteins expressed in HEK cells for the ACE2-binding assay. SDS-PAGE gels showing purity of IFNβ-ACE2 (**E**), sACE2(18-740) (**F**), sACE2(18-611) (**G**), and IFN-β (**H**) proteins.

**Mode of action and design of IFNβ-ACE2 (Figure 1).** Based on the standard mode of administration, IFN-β alone does not bind to the surface of virions, which leads to diffuse and non-targeted antiviral action (Figure 1A, top). In contrast, IFNβ-ACE2 is designed to decorate infectious virions with a surface array of IFN-β to preemptively target and upregulate antiviral activity in host cells prior to viral infection (Figure 1A, bottom). The fusion protein prototype consisted of the antiviral cytokine IFN-β covalently linked to sACE2, the main host receptor for SARS-CoV, SARS-CoV-2, and NL63 (Figure 1B). We expressed IFNβ-ACE2, recombinant IFN-β, sACE2(18-611), and sACE2(18-740) in stably transfected HEK cell lines (Figure 1B,C). Protein purification yielded 1.5–2 mg of protein per 500 mL of culture supernatant. Figure 1E–H show purity of each protein on SDS-PAGE gels. Lastly, we created an HEK cell line that expressed the full-length SARS-CoV-2 protein (D614G) (HEK-Spike) and a control HEK cell line that expressed the full-length CD25 (HEK-control) (Figure 1D).

The binding of soluble IFNβ-ACE2 to the virion-surface Spike protein is dependent on affinity, the relative ligand concentrations, and the cooperative induction/availability of the Spike protein in the ‘open’ conformation. At low IFNβ-ACE2 concentrations, a hypothetical plot showing the distribution of virions based on the ranked percentage of Spike occupancy per virion would predictably show a strong right/positively skewed distribution, where most virions exhibit low IFNβ-ACE2 occupancy of Spike on a per virion basis. At intermediate IFNβ-ACE2 concentrations, this plot would show a symmetric bell-shaped distribution, where most virions exhibited approximately 50% occupancy of Spike on a per virion basis. At high IFNβ-ACE2 concentrations, this plot would show a strong left/negatively skewed distribution, where most virions exhibit a high occupancy of Spike on a per virion basis.

The low IFNβ-ACE2 concentration range represents the most feasible pharmacologic delivery scenario, wherein non-saturating IFNβ-ACE2 concentrations in the picomolar/low nanomolar range can be achieved with high feasibility via standard delivery modalities including nebulized delivery of aerosols. These concentrations should provide effective therapeutic action based on the postulate that IFN-β directly anchored to the virion surface mediates the dominant anti-viral action of this platform independently of neutralization. Thus, in the most realistic scenarios, virions will be partly coated with IFNβ-ACE2, such that these virions may trigger IFN-β antiviral pathways concurrently with virus–host cell fusion and initial viral invasion. In this scenario, the major question is whether the kinetics of the IFN-β antiviral response can outpace a concomitant infective process to abort infection and provide protective innate immunity.

**Figure 2:**
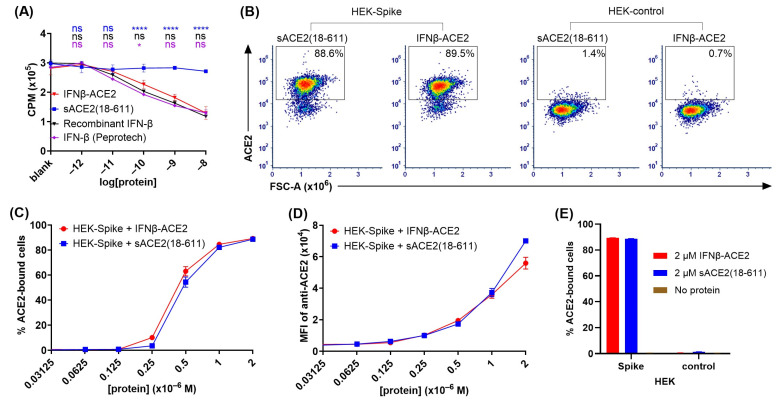
**The IFN-β and sACE2 domains of IFNβ-ACE2 exhibited predicted bioactivities.** (**A**) To assay the IFN-β domain, TF-1 cells were incubated with GM-CSF and either IFN-β, sACE2(18-611), or IFNβ-ACE2 then pulsed with [^3^H]thymidine during the last 24 h of a 3-day culture. The *y*-axis represents counts per minute (CPM), and error bars represent the SD. Statistical significance was analyzed by use of two-way ANOVA with Tukey’s multiple comparisons test comparing the three control groups to the IFNβ-ACE2 treatment group at each concentration (ns nonsignificant, * *p* < 0.05, **** *p* < 0.0001). (**B**–**E**) To assay the ACE2 domain, HEK-Spike or HEK-control cells were incubated with designated concentrations of either sACE2(18-611) or IFNβ-ACE2 for 1 h at 4 °C. After washing, cells were stained with AF647-conjugated anti-human ACE2 antibody for 1 h at 4 °C. Cells were analyzed for ACE2 binding by flow cytometry. (**B**) Viable, single, live, and GFP^+^ stably transfected cells (parental gate representing all cells in plot) were subgated to show the ACE2^+^ subset. Representative dot plots show binding of either sACE2(18-611) or IFNβ-ACE2 (2 μM each) to HEK-Spike or HEK-control cells. (**C**) Shown are percentages of ACE2^+^ HEK-Spike cells (ACE2^+^ gate/parental gate). (**D**) The MFIs of anti-ACE2 fluorescence are shown for the parental gate. (**E**) Bar graphs show mean percentages of HEK-Spike or HEK-control cells bound to ACE2 (ACE2^+^ gate/parental gate). Each data point represents the mean value (n = 2), and error bars represent SD. These data are representative of three independent experiments.

**The IFN-β domain of IFNβ-ACE2 had anti-proliferative activity comparable to that of the IFN-β control proteins (Figure 2A).** A central question was whether the individual domains of IFNβ-ACE2 exhibited predicted domain-specific activities. To assess the IFN-β domain, we used an anti-proliferative assay to compare IFNβ-ACE2 to control proteins including (a) human IFN-β (Peprotech), (b) human recombinant IFN-β protein, and (c) human recombinant sACE2(18-611) (Figure 2A). The IFNβ-ACE2 and the control proteins were cultured at designated concentrations with GM-CSF-stimulated TF-1 cells for 3 days at 37 °C, and [^3^H]thymidine incorporation was measured on the third day. As shown in Figure 2A, IFNβ-ACE2 exhibited potent anti-proliferative activity that closely approximated the inhibitory activities of the two IFN-β control proteins. Conversely, sACE2 lacked anti-proliferative activity. These data showed that the IFN-β domain of IFNβ-ACE2 retains potent interferon activity capable of inhibiting proliferative responses.

**The sACE2 domain of IFNβ-ACE2 specifically bound the transmembrane SARS-CoV-2 Spike protein (Figure 2B–E).** To assess the sACE2 domain, ACE2 binding to HEK-Spike was compared to HEK-control cells. HEK-Spike or HEK-control cells (100,000 cells/tube) were incubated with designated concentrations of either sACE2(18-611) or IFNβ-ACE2 for 1 h at 4 °C. After washing, cells were stained with AF647-conjugated anti-human ACE2 antibody and were analyzed for ACE2 binding by flow cytometry. Figure 2B shows representative dot plots of ACE2 binding of 2 μM sACE2(18-611) or IFNβ-ACE2 to HEK-Spike and HEK-control cells. IFNβ-ACE2 had similar binding to HEK-Spike as sACE2(18-611), as indicated by percentages of cells in the ACE2^+^ gate (Figure 2C) and by the anti-ACE2 MFI (Figure 2D). As shown in Figure 2E, IFNβ-ACE2 and sACE2(18-611) exhibited robust binding to HEK-Spike cells but did not exhibit detectable binding to HEK-control cells. These data provide evidence that the sACE2 domain of IFNβ-ACE2 maintains robust binding to transmembrane SARS-CoV-2 Spike protein.

**Figure 3:**
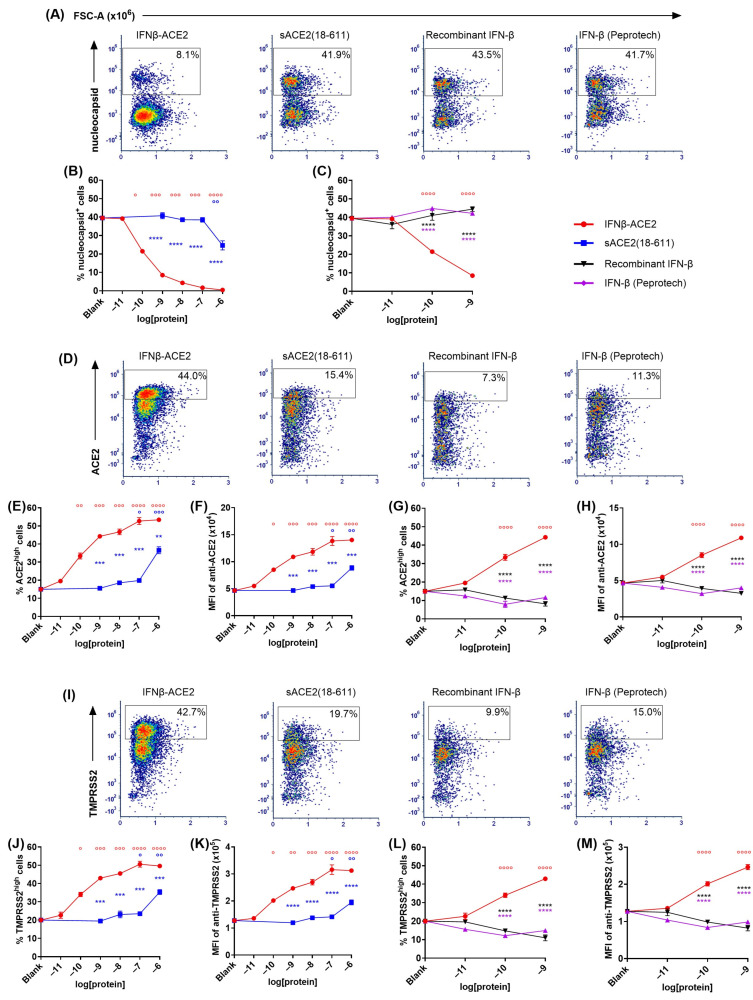
**The sACE2 domain of IFNβ-ACE2 targeted IFN-β to the surface of NL63.** NL63 was incubated at 4 °C with designated concentrations of IFNβ-ACE2, sACE2(18-611), recombinant IFN-β, or IFN-β (Peprotech). After a 1 h incubation, NL63 was washed of any unbound protein using 300kD centrifugal filters. NL63-protein complexes were then added to Vero E6-TMPRSS2-T2A-ACE2 cultures (100 μL) in a 96-well plate. The cells were harvested after a 2-day incubation at 33 °C, stained with LIVE/DEAD Fixable Blue Dead Cell Stain, and then surface-labeled with FITC-conjugated anti-human ACE2 and PE-conjugated anti-human TMPRSS2. After fixation and permeabilization, cells were stained with AF647-conjugated rabbit anti-NL63 nucleocapsid antibody. Cells were then analyzed for viral infection by flow cytometry. Cells were gated on viable, single, and live cells (parental gate) before subgating on nucleocapsid^+^, ACE2^high^, or TMPRSS2^high^ cells. Shown are representative dot plots ((**A**,**D**,**I**), *x*-axis = FSC-A as in (**A**)) when NL63 was incubated with 1 nM IFNβ-ACE2 or controls. The IFNβ-ACE2 versus sACE2(18-611) groups were compared based on percentages of nucleocapsid^+^ cells (**B**), percentages of ACE2^high^ cells (**E**), MFI of anti-ACE2 staining (**F**), percentages of TMPRSS2^high^ cells (**J**), and MFI of anti-TMRSS2 staining (**K**). The IFNβ-ACE2 versus IFN-β groups were compared based on percentages of nucleocapsid cells (**C**), percentages of ACE2^high^ cells (**G**), MFI of anti-ACE2 staining (**H**), percentages of TMPRSS2^high^ cells (**L**), and MFI of anti-TMPRSS2 staining (**M**). Cell percentages were calculated by dividing events in the positive subgate by the parental gate, and MFI values represent all events in the parental gate. Each data point represents the mean value (n = 2), and error bars represent SD. Statistical significance comparing IFN-β and ACE2 treatment groups to the IFNβ-ACE2 treatment group at each concentration was analyzed by use of two-way ANOVA with Tukey’s multiple comparisons test (** *p* < 0.01, *** *p* < 0.001, **** *p* < 0.0001). Statistical significance comparing each protein group at each concentration to blank was analyzed by use of two-way ANOVA with Tukey’s multiple comparisons test (° *p* < 0.05, °° *p* < 0.01, °°° *p* < 0.001, °°°° *p* < 0.0001). These data are representative of three independent experiments.

**Figure 4:**
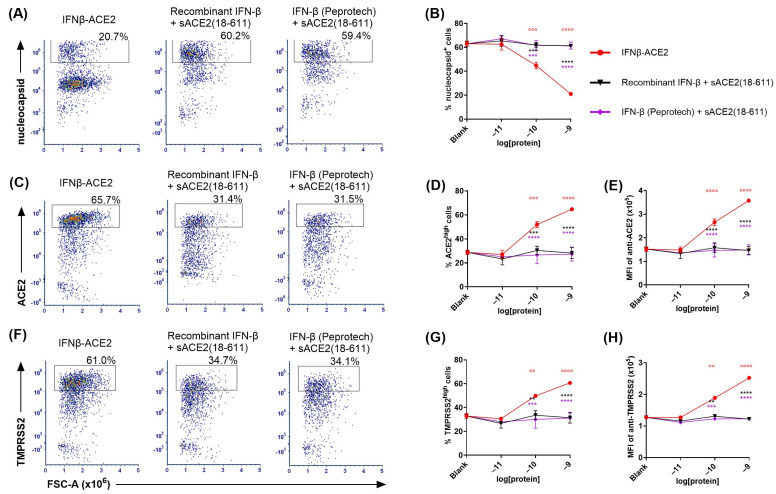
**The covalent linkage of IFN-β and ACE2 was required for IFN-β targeting to NL63.** NL63 was incubated at 4 °C with either IFNβ-ACE2 or the unlinked combination of sACE2(18-611) and IFN-β. After a 1 h incubation, NL63 was repeatedly washed with 300kD centrifugal filters to remove proteins that lacked binding to virions. The retentates, which included virions and virion-bound proteins, were added to Vero E6-TMPRSS2-T2A-ACE2 cells in a 96-well plate. Cells were harvested after a 2-day incubation at 33 °C and stained with LIVE/DEAD Fixable Blue Dead Cell Stain. Cells were surface-stained with PE-conjugated mouse anti-human TMPRSS2 and FITC-conjugated mouse anti-human ACE2. After fixation and permeabilization, cells were stained with AF647-conjugated rabbit anti-NL63 nucleocapsid antibody. Cells were analyzed for viral infection by flow cytometry. Viable, single, and live cells in the parental gate were subgated as the nucleocapsid^+^ subset (**A**), the ACE2^high^ subset (**C**), and the TMPRSS2^high^ subset (**F**) as shown for the 1 nM concentration value. Shown are the percentages of nucleocapsid^+^, ACE2^high^, and TMPRSS2^high^ subsets together with the respective MFI values (**B**,**D**,**E**), and (**G**,**H**), respectively. Cell percentages were calculated by dividing the events in the subset-positive/high subgate by those in the parental gate. MFI values were gated on all viable, single, and live cells (i.e., cells in the parental gate). Each data point represents the mean value (n = 2), and error bars represent SD. Statistical significance of the IFNβ-ACE2 versus the ‘IFN-β + sACE2’ treatment group at each concentration was analyzed by use of two-way ANOVA with Tukey’s multiple comparisons test (** *p* < 0.01, *** *p* < 0.001, **** *p* < 0.0001). Statistical significance was also assessed for treatment groups at each concentration compared to the ‘blank’ control via two-way ANOVA with Tukey’s multiple comparisons test (°° *p* < 0.01, °°° *p* < 0.001, °°°° *p* < 0.0001). These data are representative of three independent experiments.

**The sACE2 domain of IFNβ-ACE2 targeted IFN-β to the surface of NL63 (**Figure 3 and Figure 4**).** The primary question addressed by this study was whether the sACE2 domain of IFNβ-ACE2 targets IFN-β to the surface of NL63 to mediate preemptive antiviral activity. To assess this question, we ran flow cytometric assays to measure intracellular nucleocapsid expression as a measure of viral infection. NL63 (MOI of 0.1) was incubated for 1 h with either IFNβ-ACE2, sACE2(18-611), IFN-β (Peprotech), or recombinant IFN-β. The virus plus protein preparations were then subjected to repeated centrifugal ultrafiltration through a 300 kDa MWCO filter to filter unbound protein into the filtrate, whereas stable virus–protein complexes were retained in the retentate. ‘Virus-washed’ NL63 and/or NL63-protein complexes in the retentate were subsequently seeded onto Vero E6-TMPRSS2-T2A-ACE2 cells. After a 48 h incubation, cells were harvested, stained, and analyzed by flow cytometry to measure viral infection. Flow cytometric analysis showed that infected versus uninfected cells presented as qualitatively distinct populations, with virally infected cells presenting as a distinct nucleocapsid^+^ phenotype whereas uninfected cells were distinctly nucleocapsid^-^ (Figure 3A). IFNβ-ACE2 at an initial concentration range of 100 pM–1 μM bound to NL63 and was retained as virion-bound IFNβ-ACE2 to cause inhibition of NL63 nucleocapsid production (Figure 3B,C). The mechanism of inhibition was attributed largely to the antiviral activity of the IFN-β domain rather than to ACE2-mediated neutralization, because sACE2(18-611) alone only exhibited inhibition at the highest concentration tested (1 μM) (Figure 3B). Rather, the sACE2 domain of IFNβ-ACE2 was needed to anchor IFN-β to the virion surface because neither IFN-β preparation alone had antiviral activity, presumably because IFN-β did not independently bind the virus and therefore was lost in the filtrate (Figure 3C). The distinction of nucleocapsid^+^ versus nucleocapsid^-^ subsets as infected versus uninfected subsets was validated in that the nucleocapsid^+^ subset corresponded to an ACE2^low^ and TMPRSS2^low^ phenotype, and vice versa, which corresponds with the downregulation of the ACE2 host receptor and the TMPRSS2 coreceptor upon infection (Figure 3D–M). Based on ACE2 and TMPRSS2 expression, IFNβ-ACE2 robustly inhibited infection across a concentration range of 100 pM to 1 μM, whereas sACE2(18-611) alone inhibited infection only at the highest concentration tested (1 μM) (Figure 3E,F,J,K), and both IFN-β preparations alone lacked detectable antiviral activity (Figure 3G,H,L,M). Overall, these data reveal that IFNβ-ACE2 binds to virions to mediate robust antiviral interferon activity as evidenced by parallel concentration–response curves for the three infected nucleocapsid^+^, ACE2^low^, and TMPRSS2^low^ phenotypes. These data support the hypothesis that IFNβ-ACE2 provides specific targeting of interferon activity to the virion surface, which is the most proximal site of viral infectivity.

A remaining question was whether the covalent linkage between IFN-β and sACE2 in IFNβ-ACE2 was required for the enhanced antiviral efficacy. These experiments thereby addressed the formal possibility that unlinked sACE2 and IFN-β could act synergistically as independent molecules directly on the virus to inhibit viral infectivity, via unanticipated mechanisms. To address this question (Figure 4), we used the ‘virus-washed’ in vitro assay to compare IFNβ-ACE2 to the unlinked combination of equimolar sACE2(18-611) and IFN-β, with nucleocapsid expression (Figure 4A,B) coupled with ACE2 and TMPRSS2 downregulation (Figure 4C–E and 4F–H, respectively) as measures of infection. As predicted, these data showed that the covalent linkage between IFN-β and sACE2 was critical for targeting. These data support the hypothesis that sACE2 binds the Spike protein to anchor IFN-β on the virion surface to ‘flip’ the virus to a positive interferon-signaling entity.

**Figure 5:**
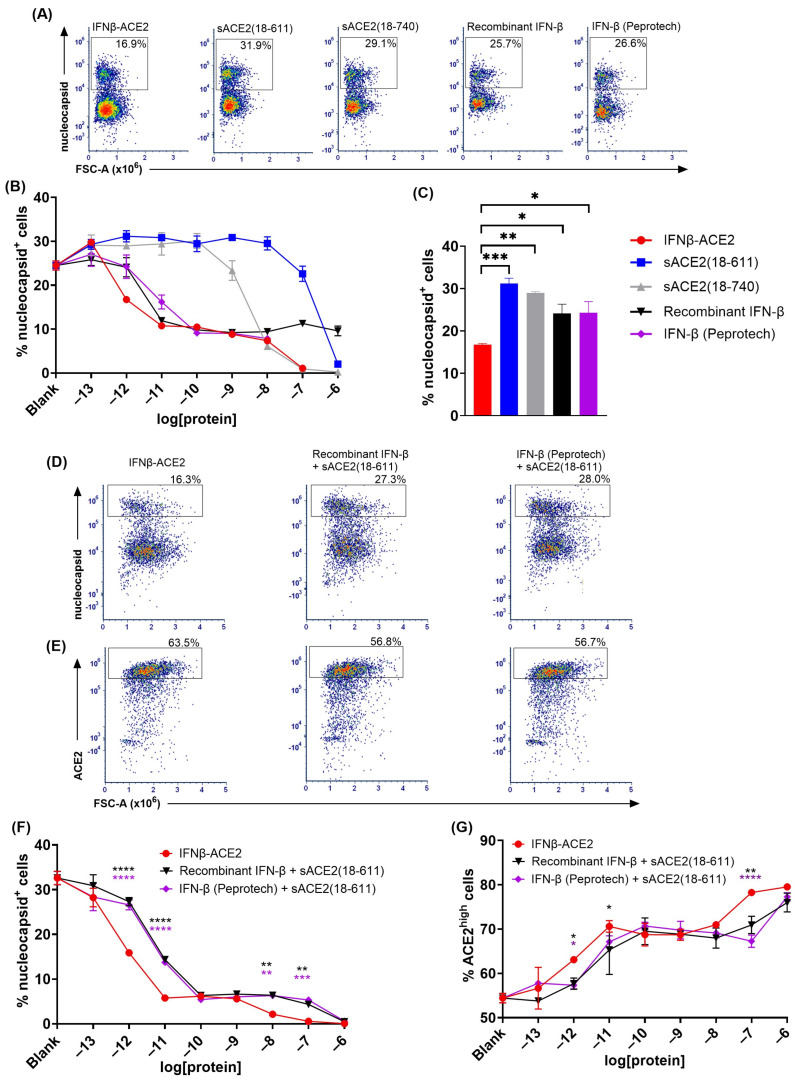
**In a non-washed in vitro infection system, IFNβ-ACE2 exhibited enhanced antiviral activity compared to IFN-β alone, ACE2 alone, or the unlinked combination.** NL63 was incubated for 1 h at 4 °C with either IFNβ-ACE2, sACE2(18-611), sACE2(18-740), recombinant IFN-β, IFN-β (Peprotech), or the unlinked combination of sACE2(18-611) and IFN-β. In contrast to experiments shown in Figure 3 and Figure 4, we omitted the virus-washing step. The NL63 + protein mixtures were added to Vero E6-TMPRSS2-T2A-ACE2 cells in a 96-well plate. The cells were harvested after a 2-day incubation at 33 °C and stained with LIVE/DEAD Fixable Blue Dead Cell Stain. Cells were surface-stained with FITC-conjugated mouse anti-human ACE2, were fixed and permeabilized, and then were intracellularly stained with AF647-conjugated rabbit anti-NL63 nucleocapsid antibody. Cells were then analyzed for viral infection by flow cytometry. Cells gated as viable, single, and live cells (parent gate) were subgated to define nucleocapsid^+^ and ACE2^high^ subsets. Shown (**A**,**D**) are representative dot plots showing percentages of the nucleocapsid^+^ subset at the 1 pM concentration. Shown (**B**,**F**) are the percentages of the nucleocapsid^+^ subset for each group over concentrations ranging from 100 fM to 1 μM. Bar graph (**C**) shows mean percentage values of nucleocapsid^+^ cells at the 1 pM concentration. Shown (**E**) are representative dot plots showing percentages of the ACE2^high^ subset at the 1 pM concentration. Shown (**G**) are the percentages of ACE2^high^ subset for each group over concentrations ranging from 100 fM to 1 μM. Each data point represents the mean value (n = 2), and error bars represent SD. Statistical significance was analyzed by (**C**) one-way ANOVA with the Dunnett multiple comparisons test or (**F**,**G**) two-way ANOVA with Tukey’s multiple comparisons test comparing the unlinked combination of IFN-β and ACE2 treatment groups to the IFNβ-ACE2 treatment group at each concentration (* *p* < 0.05, ** *p* < 0.01, *** *p* < 0.001, **** *p* < 0.0001). These data are representative of three independent experiments.

**IFNβ-ACE2 had potentiated antiviral activity compared to IFN-β and/or sACE2 in a non-washed in vitro infection system (Figure 5).** The rationale for a ‘virus-washed’ system (Figure 3 and Figure 4) was to assess the antiviral action of virion surface-bound IFNβ-ACE2 and sACE2. However, this system did not assess soluble IFN-β action because soluble IFN-β did not independently bind the virus and therefore was not retained in the retentate. Thus, comparison of ‘virus-washed’ systems to ‘non-washed’ in vitro infection systems was needed to compare virus-targeted and virus-non-targeted IFN-β/IFNAR interactions. That is, the ‘virus-washed’ system solely reveals the action of virion surface IFN-β, whereas the non-washed system combines the activities of virus-anchored IFN-β and virus-non-targeted ‘free’ IFN-β. The prediction is that virus-mediated targeting of IFN-β will be less apparent when all reagents are present within the confines of the 96-well 100 μL volume.

In non-washed infection systems, virus and reagents were incubated together for 1 h then added directly to the well without a virus-washing step. That is, NL63 (MOI of 0.1) was incubated with IFNβ-ACE2, sACE2(18-611), sACE2(18-740), recombinant IFN-β, IFN-β (Peprotech), or the unlinked combination of sACE2(18-611) and IFN-β. After a 1 h incubation at 4 °C, the NL63 and protein mixtures were seeded (without washing virus) onto Vero E6-TMPRSS2-T2A-ACE2 cells. After a 48 h incubation, cells were harvested, stained, and analyzed for infection by flow cytometry. Figure 5A,D show the nucleocapsid^+^ gate (infected cells) whereas Figure 5E shows the ACE2^high^ gate (noninfected cells). Several results were noted. (a) IFNβ-ACE2 exhibited a biphasic mechanism of antiviral activity as shown by intracellular staining of nucleocapsid (Figure 5B,F) and surface staining of ACE2 (Figure 5G). The first phase of IFNβ-ACE2 inhibition was evident in the 100 fM–100 pM concentration range. This highly potent antiviral mechanism represented the antiviral activity of the IFN-β domain, which coincided with the antiviral activity of the two free IFN-β preparations. (b) The second phase of IFNβ-ACE2 antiviral inhibition was evident in the 10 nM–1 μM concentration range. This inhibitory mechanism represented sACE2-mediated viral neutralization, which coincided with the antiviral sensitivity of sACE2. Thus, IFN-β-mediated antiviral activity was more potent by several orders of magnitude compared to the neutralizing activity of the monomeric sACE2(18-611) and the dimeric sACE2(18-740). (c) IFNβ-ACE2 exhibited antiviral activity that was 10-fold more potent than IFN-β. This finding supported the possibility that targeting of IFNβ-ACE2 to the viral surface potentiated antiviral activity in non-washed in vitro infection systems. (d) IFNβ-ACE2 appeared to have neutralization activity that was more potent than the control sACE2(18-611) protein. This finding shows that physical coupling of sACE2 to IFN-β does not impair sACE2 neutralizing activity. (e) Dimeric sACE2(18-740) had potentiated neutralization activity compared to monomeric sACE2(18-611) by approximately two orders of magnitude (Figure 5B). These data provide evidence that multimeric sACE2 proteins exhibit more stable binding interactions with NL63, which probably reflects increased binding avidity to Spike trimers. (f) These data complement the ‘virus-washed’ experiments (Figure 3 and Figure 4), which showed IFNβ-ACE2 had antiviral activity with 100 pM sensitivity, which is four orders of magnitude lower than the 1 μM sensitivity of sACE2(18-611)-mediated neutralization. These data reveal the predominant mechanism wherein the sACE2 domain provides an anchoring function that decorates Spike with protruding IFN-β molecules. Conversely, direct sACE2-mediated neutralization appears to be an inhibitory mechanism that was evident only at high concentrations.

**Figure 6:**
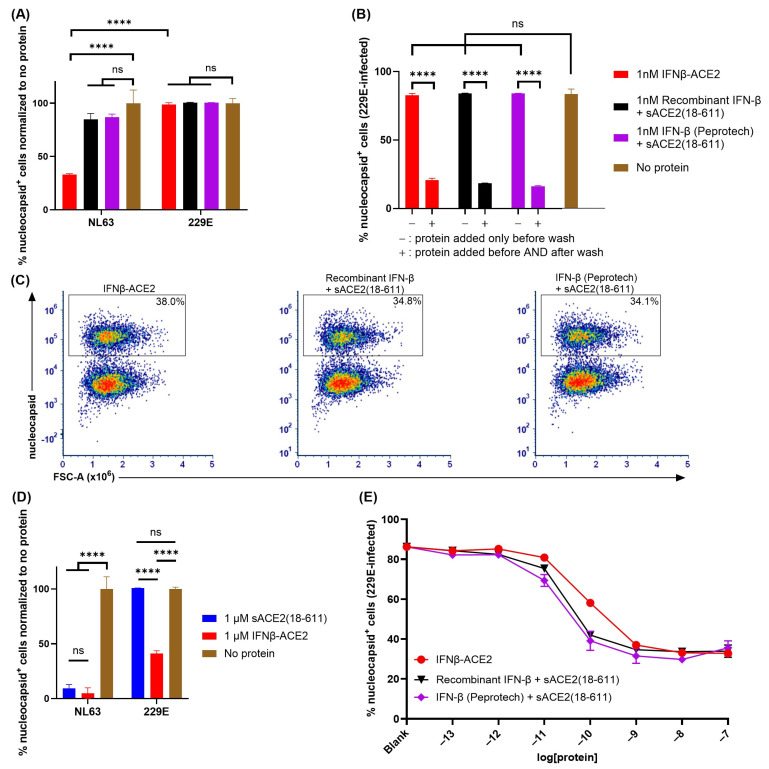
**IFNβ-ACE2 exhibited virus-specific targeting in accordance with viral host receptor specificity**. The NL63 or 229E viruses were incubated at 4 °C with either IFNβ-ACE2, sACE2(18-611), IFN-β, or the unlinked combination of IFN-β and sACE2(18-611). After a 1 h incubation, the virus + protein mixtures were washed, and the retentate containing virion–protein complexes was used for infection of Vero E6-TMPRSS2-T2A-ACE2 or A549 cells, respectively, in a 96-well plate (**A**,**B**). After the washing step, IFNβ-ACE2 or the unlinked combination of IFN-β and sACE2(18-611) were directly added to designated groups (**B**). Alternatively, the virus + protein mixtures were not subjected to a virus-washing step and the mixtures were used for infection of the respective host cells (**C**–**E**). The cells were harvested after a 2-day incubation and stained with LIVE/DEAD Fixable Blue Dead Cell Stain. After fixation and permeabilization, Vero E6-TMPRSS2-T2A-ACE2 cells were stained with AF647-conjugated rabbit anti-NL63 nucleocapsid antibody and A549 cells were stained with AF647-conjugated rabbit anti-229E nucleocapsid antibody. Cells were then analyzed for viral infection by flow cytometry. Cells were gated on viable, single, and live cells before subgating on nucleocapsid^+^ cells. Shown (**A**) are the percentages of nucleocapsid^+^ cells for each treatment group normalized to the ‘no protein’ control group (1 nM concentrations). (**B**) Bar graph shows mean percentages of nucleocapsid^+^ 229E-infected cells when proteins were or were not added after the washing step (1 nM concentrations). Shown (**C**) are representative dot plots including percentages of nucleocapsid^+^ 229E-infected cells (1 nM concentrations). Shown (**D**) are the percentages of nucleocapsid^+^ cells for each treatment group normalized to the ‘no protein’ group for each virus (1 μM concentrations). Shown (**E**) are the percentages of nucleocapsid^+^ 229E-infected cells for each treatment group at designated concentrations (100 fM to 100 nM). Each data point represents the mean value (n = 2), and error bars represent SD. Statistical significance was analyzed by use of two-way ANOVA with Tukey’s multiple comparisons test comparing the IFN-β and/or ACE2 treatment groups to the IFNβ-ACE2 treatment group at each concentration (unless otherwise noted in the figure) (ns nonsignificant, **** *p* < 0.0001). Experiments shown are representative of three independent experiments.

**The sACE2 domain of IFNβ-ACE2 targeted IFN-β to NL63 but not to 229E (Figure 6).** A central question focused on the virus-targeting specificity of IFNβ-ACE2. An important question was whether the sACE2 domain of IFNβ-ACE2 also targeted IFN-β to the surface of HCoV 229E which binds human aminopeptidase N (APN) rather than ACE2 as the host receptor. To address this question, we performed ‘virus-washing’ experiments with HCoVs NL63 (MOI of 0.1) and 229E (MOI of 0.1) by incubating 1 nM IFNβ-ACE2 or the unlinked combination of 1 nM sACE2(18-611) and 1 nM IFN-β with NL63 or 229E. After 1 h, the viruses were repeatedly washed via the 300kDa MWCO Nanosep centrifugal filters. Ultrafiltration retentates containing NL63-protein complexes or 229E-protein complexes were then seeded onto Vero E6-TMPRSS2-T2A-ACE2 or A549 cells, respectively, with or without re-addition of protein (IFNβ-ACE2 or recombinant protein controls). After a 48 h incubation, the cells were harvested, stained, and analyzed by flow cytometry. The central finding was that IFNβ-ACE2 exhibited antiviral activity against NL63 but had no antiviral effect against 229E (Figure 6A). The unlinked combination of sACE2(18-611) and IFN-β had no effect on either virus (Figure 6A,B) because sACE2(18-611) is sub-neutralizing at this concentration (1 nM) and the IFN-β was presumably lost in the filtrate during the virus-washing procedure. The antiviral activity of IFN-β on 229E was confirmed in control groups wherein IFNβ-ACE2 or the IFN-β + sACE2(18-611) proteins were re-added to control host cell cultures after the virus-washing procedure (Figure 6B). These control groups validated the IFN-β-mediated antiviral susceptibility of 229E. These data show that the sACE2 domain specifically binds to NL63 but not 229E in accordance with Spike host receptor specificity. Thus, the sACE2 domain is a specific virus-targeting modality that reflects the host receptor specificity of the viral Spike protein.

Similar findings were noted in a non-washed in vitro infection system where 1 μM sACE2(18-611) or IFNβ-ACE2 were incubated with NL63 (MOI of 0.1) or 229E (MOI of 0.1) and then added (without virus wash) to Vero E6-TMPRSS2-T2A-ACE2 or A549 cells, respectively. The main finding was that sACE2(18-611) robustly inhibited NL63 infectivity but was without effect on 229E infectivity, which confirms the specificity of sACE2 for NL63 but not 299E (Figure 6D). Also, IFNβ-ACE2 fully inhibited NL63 infectivity, which represented the covalently linked action of sACE2-mediated neutralization and anchoring and IFN-β-mediated antiviral activity. In contrast, IFNβ-ACE2 only partially inhibited 229E infectivity, which reflected a unilateral IFN-β-mediated antiviral activity that plateaued at a nadir of approximately 40% infection in the 1–100 nM concentration range (Figure 6C–E). The overlapping infection curves indicate that IFNβ-ACE2 did not inherently have more antiviral activity compared to the ‘IFN-β + sACE2’ control proteins, such that the IFNβ-ACE2 covalent linkage was not important for 229E. Overall, these findings support the concept that highly specific interactions of the sACE2 domain with an ACE2-specific viral Spike glycoprotein appears critical for the anchoring of IFNβ-ACE2 antiviral activity directly to the surfaces of pathogenic viruses in ACE2-viral tropism.

## 4. Discussion

*IFNβ-ACE2 is a prototype for a new modular class of antiviral therapeutics*: The SARS-CoV-2 pandemic highlighted the need for modular antiviral platforms that can be repurposed to target emerging viral pathogens. The main advantage of the IFNβ-ACE2 fusion protein is that the physical linkage of sACE2 and IFN-β provides synergistic benefits because the virus/IFNβ-ACE2 complex creates an IFN-β array at the viral surface such that robust IFN-β signaling and upregulation of antiviral innate defenses invariably precede cellular infection (Figure 1). This innovative concept has no parallel in the contemporary landscape of antiviral therapeutics. Based on the trimeric structure of the Spike protein, each Spike protein at the virion surface can ‘present’ as many as three IFN-β proteins to provide an outer shell of IFN-β that activate IFNAR signaling upon contact of the virus with a host cell. This targeted presentation of IFN-β at the viral surface is qualitatively different from subcutaneous or intravenous IFN-β administration, which leads to systemic distribution of IFN-β coupled with highly diffuse antiviral action, low IFN-β concentrations at the site of infection, and a primary impact on non-target cell types irrelevant to the viral infection. Importantly, this modular antiviral platform can be modified by replacement of the sACE2 domain with other viral host receptors to target alternative spectrums of viral pathogens. Likewise, the IFN-β effector domain can be replaced with alternative effector domains to engage other antiviral defenses that may be more applicable to selected viral pathogens.

*Rationale for use of sACE2 as an anti-inflammatory, antiviral mediator*: The sACE2 domain has both anti-inflammatory and antiviral activity. The sACE2 domain is a major anti-inflammatory regulatory node of the renin–angiotensin system, in that ACE2 hydrolyzes the inflammatory angiotensin II hormone to the homeostatic angiotensin 1–7 modulator [18]. ACE2-mediated anti-inflammatory activities may ameliorate diseases such as acute respiratory distress syndrome (ARDS), acute lung injury (ALI), and SARS [31,32,33,34]. Conversely, downregulation of transmembrane ACE2 in SARS-CoV- and SARS-CoV-2-infected cells is thought to exacerbate disease [32,35,36,37,38,39,40]. In addition to anti-inflammatory activity, the sACE2 domain also has antiviral applications, because sACE2 neutralizes the Spike protein of HCoV NL63, SARS-CoV, and SARS-CoV-2 and thereby inhibits infection in vitro [14,34,41,42].

Viruses may exhibit the evolutionary adaptation of reprogramming infected cells to shed host receptor and/or coreceptors to preclude superinfection. Virus-induced host receptor shedding causes release of the respective ectodomains into extracellular fluids and the circulation such that infection severity may correlate with circulating levels of receptor/coreceptor ectodomains. This paradigm may apply to ACE2-tropic coronaviruses because the severity of infection appears to correlate with circulating levels of sACE2, which is used as a biomarker of infection. However, circulating sACE2 retains enzymatic activity and continues to produce anti-inflammatory angiotensin derivatives that may counteract virally induced immunopathogenesis. Circulating sACE2 may also mediate viral neutralization. Although circulating levels of sACE2 may correlate with COVID-19 pathogenesis, circulating sACE2 may be compensatory and serve to alleviate immunopathogenesis and facilitate disease remission. Thus, the transmembrane ACE2 host receptor, which is required for pathogenesis, may diametrically differ from the shed sACE2 ectodomain, which may act to reverse pathogenesis. In support, a phase 2 trial for COVID-19 showed that intravenous sACE2 significantly improved mechanical ventilator-free days and reduced viral RNA load compared to placebo (APEIRON, clinicaltrials.gov ID NCT04335136). Additionally, intravenous sACE2 led to a decrease in pro-inflammatory angiotensin II and an increase in anti-inflammatory angiotensin 1–7 and angiotensin 1–5 (APEIRON, clinicaltrials.gov ID NCT04335136). In a phase I study, aerosolized sACE2 was found to be safe and well-tolerated, with no dose-limiting toxicities and no detection of anti-ACE2 antibodies (APEIRON, clinicaltrials.gov ID NCT05065645) [43]. Thus, in addition to the sACE2-mediated targeting of IFNβ-ACE2 to the virion surface, the sACE2 domain may provide anti-inflammatory activity to improve overall therapeutic efficacy.

*Rationale for use of sACE2 rather than an anti-Spike antibody as a targeting moiety*: As an alternative to the IFNβ-ACE2 platform, fusion proteins that couple IFN-β to high-affinity anti-Spike antibodies/nanobodies may more efficiently target IFN-β to surfaces of selected pathogenic viruses, simply because anti-Spike modalities may have higher affinity than sACE2 for Spike glycoproteins. Nonetheless, the choice of ACE2 as the targeting vehicle has several major advantages over anti-Spike antibodies. Namely, the IFNβ-ACE2 platform targets diverse viral species, including NL63, SARS-CoV, and SARS-CoV-2, and all variants of these viral species. Thus, the IFNβ-ACE2 platform is impervious to immune evasion via immune-selected variant evolution. The IFNβ-ACE2 platform may also target future pandemic viral pathogens that use ACE2 as the primary host receptor by a cross-reactive mechanism that would preclude continual emergence of new pathogenic variants. Conversely, an IFNβ-anti-Spike platform would likely target a limited number of variants of one viral species and would have to be reinvented and gain new regulatory approvals seasonally for each new emergent viral variant. Given that the IFNβ-ACE2 affinity for Spike is adequate to achieve robust antiviral activity (Figure 3 and Figure 4), the IFNβ-ACE2 platform appears to have compelling advantages over an IFNβ-anti-Spike platform.

*Rationale for use of type I interferons as the effector moiety*: Over the past several decades, IFN-Is have proven to be safe and effective as FDA-approved biologics for a broad spectrum of disease classes, including cancer, infectious diseases (e.g., hepatitis B and C), and autoimmune diseases (e.g., multiple sclerosis) [44,45,46,47]. IFN-Is were extensively tested in COVID-19 and were uniformly found to be safe without adverse consequences, and multiple studies provided suggestive evidence of clinical efficacy [48,49,50,51,52,53,54,55]. For example, a phase 2 trial showed that inhaled pulmonary administration of IFN-β was efficacious in treating SARS-CoV-2-infected patients, as shown by accelerated recovery and a 79% decrease in progression to severe disease or death (Synairgen, clinicaltrials.gov ID NCT04385095) [52]. A phase 3 trial showed that nebulized IFN-β trended towards reducing relative risk of progression to severe disease or death within 35 days compared to patients who received placebo, especially in high-risk patient subgroups. However, there was no significant difference in the time to recovery and time to hospital discharge (primary endpoints) (Synairgen, isrctn.com ID ISRCTN85436698) [55]. Additionally, subcutaneous administration of IFN-β in the World Health Organization Solidarity Trial did not show clinical efficacy [50], which raised the question of whether subcutaneous IFN-I is an optimal administration route. From a safety standpoint, these clinical studies showed that administration of IFN-Is was well-tolerated, lacked pro-inflammatory action, and did not exacerbate disease. Given that IFN-I administration via parenteral or mucosal routes is associated with inconsistent or modest efficacy, new strategies are needed for dose-sparing, targeted delivery of interferon-based drugs to improve efficacy. It is informative to compare clinical application of IFN-I to the endogenous innate production of IFN-I in that the innate immune system locally produces IFN-I at sites of viral infection. Thus, an important clinical goal is to administer IFN-I via strategies in line with the specificity and locality of the innate immune response. As SARS-CoV-2 inhibits IFN-I production, the IFNβ-ACE2 targeting strategy may fulfill these criteria by introducing IFN-β directly to the site of infection by binding to ACE2-tropic viruses, including emergent coronaviruses that represent a clear global threat for future pandemics.

*Interpretation of the ‘virus-washing’ methodology*: The main question of this study is whether anchoring IFN-β to the virion surface enables strong antiviral activity. Although the ‘virus-washing’ experiments are unconventional, the ‘virus-washing’ approach represented a direct means to test our hypothesis. Importantly, the ‘virus-washing’ methodology provided the key experimental data for the conclusion that IFNβ-ACE2 mechanistically targeted IFN-β to confer high anti-viral efficacy to the exact physical interface of infection. The comparison of ‘virus-washed’ (Figure 3 and Figure 4) and ‘non-washed’ (Figure 5) systems also enabled comparison of virus-anchored IFN-β (targeted) versus virus-anchored IFN-β mixed with free IFN-β (non-targeted) systems. In comparison to IFN-β, IFNβ-ACE2 had superlative antiviral activity in targeted systems and had advantageous antiviral activity in non-targeted systems. That is, in a non-targeted system, IFNβ-ACE2 had 3–10-fold enhanced potency compared to the unlinked combination of IFN-β and sACE2 (Figure 5). The EC_50_ of IFNβ-ACE2 was ~1 pM, whereas the EC_50_ of IFN-β or the unlinked combination of IFN-β and sACE2(18-611) was ~10 pM. In conclusion, IFNβ-ACE2 had major advantages over IFN-β in targeted systems but also had significant advantages compared to IFN-β in non-targeted systems.

*The physiological significance represented by the ‘virus-washing’ methodology*: In this study, we compared two environments via the ‘virus-washed’ versus ‘non-washed’ virus systems. The ‘virus-washed’ system uses centrifugal fluid flow to remove free unbound proteins while retaining proteins that are directly bound to the virion surface. In non-washed conditions, all protein reagents, virions, and cells were confined to a 100 μL volume without any directional fluid flow. A central question pivots on which system best reflects the in vivo environment, given that all physiological environments are subject to constant dynamic fluid flow. Fluid flow in the lung parenchyma is driven by rhythmic ciliary beating that constantly moves fluid and mucus from the lung. Major fluidic drivers in all tissues include circulatory and/or lymphatic flow, together with many complementary drivers (osmotic, hydrostatic, oncotic, etc.). The virus-washed system was devised to address a technical question: does virion-anchored IFN-β exhibit antiviral efficacy? We believe that virus-washed systems have physiological significance, because the system features fluid flow dynamics which are central to all physiological environments. That is, IFNβ-ACE2/virus complexes will retain targeted antiviral activity regardless of in vivo fluid flow dynamics that would otherwise separate an antiviral reagent from infectious virions. Unlike IFNβ-ACE2, for example, free IFN-β will readily diffuse from infectious virus by any differential/diffusional flow kinetics. In a physiological context, fluid-flow tissue transit of virions versus soluble IFN-β differs fundamentally. Virions are more massive, are precipitable rather than soluble, are readily trapped in a web of extracellular matrix, and form extracellular long-lived infectious reservoirs upon binding cell appendages or tissue matrix components. IFN-β, in contrast, is freely diffusible and swiftly achieves broad systemic biodistribution coupled with rapid degradation. Thus, the principle of this platform is defined by the anchoring of IFNβ-ACE2 to virion surfaces coupled with pinpoint targeting of antiviral activity to the site of infection by a mechanism impervious to differential fluid flow. The tight physical association of IFNβ-ACE2 and virions and the lack of differential diffusion (Figure 3 and Figure 4) are unique features of this platform with important physiological significance.

*Is antiviral efficacy of IFNβ-ACE2 contingent upon ACE2-mediated targeting and/or ACE2-mediated neutralization?* This study shows that the sACE2 domain of IFNβ-ACE2 effectively targets IFN-β to the surface of NL63 at sub-neutralizing concentrations. Indeed, IFNβ-ACE2 exhibited biphasic inhibition curves (Figure 5) in that IFN-β-mediated antiviral activity was several orders of magnitude more potent than the neutralizing activity of sACE2. These findings support the concept that the low-zone picomolar concentration range mediates the dominant antiviral activity of IFNβ-ACE2 without the need for viral neutralization, which is only observed at much higher concentrations (Figure 3B). Based on this vantage point, the dominant mechanism of antiviral efficacy may be ACE2-mediated targeting of IFN-β to the viral surface rather than ACE2-mediated viral neutralization. Under these conditions, viruses will be partly neutralized and decorated with IFN-β. This will lead to IFN-β signaling and upregulation of the antiviral state within the cell prior to or concurrently with viral infection. Future experimentation in preclinical infection models will reveal the dose-dependent antiviral activity of IFNβ-ACE2 versus control proteins, and these experiments will reveal whether ACE2-mediated targeting alone or in combination with ACE2-mediated neutralization represents the dominant therapeutic mechanism in vivo.

*NL63 viral infection causes transmembrane ACE2 and TMPRSS2 downregulation together with intracellular nucleocapsid production*: Transmembrane TMPRSS2 plays an important role in NL63, SARS-CoV, and SARS-CoV-2 Spike protein priming and viral entry by cleaving Spike protein at the S1/S2 and the S2′ sites [17,27,56,57,58,59,60]. As shown in Figure 3D–M and Figure 4C–H, NL63 infection of Vero E6-TMPRSS2-T2A-ACE2 cells caused downregulation of ACE2 and TMPRSS2. Because IFNβ-ACE2 blocks NL63 infection, IFNβ-ACE2 also preserved normal surface levels of ACE2 and TMPRSS2. As SARS-CoV and SARS-CoV-2 infection is postulated to cause pulmonary inflammation in part by causing ACE2 downregulation, IFNβ-ACE2 may prevent inflammatory lung diseases in infected patients by blocking ACE2 downregulation and augmenting anti-inflammatory sACE2 concentrations [31,33,35,37,61]. A previous report, however, noted that NL63 infection did not decrease ACE2 expression on VeroE6 cells despite the increase in viral RNA and protein levels [35] in contrast to our study showing that NL63 infection drives decreased ACE2 expression on Vero E6-TMPRSS2-T2A-ACE2 cells. This inconsistency could be due to multiple reasons. First, we compared NL63 infection in VeroE6 and Vero E6-TMPRSS2-T2A-ACE2 cells and found that Vero E6-TMPRSS2-T2A-ACE2 cells are more susceptible to NL63 infection. As the decrease in ACE2 expression is dependent on the efficiency of NL63 infection and replication [62], this increase in infection may lead to the downregulation and/or shedding of ACE2. Second, flow cytometry is an inherently more sensitive technique than Western blots. Overall, transmembrane ACE2 and TMPRSS2 downregulation closely correlated with nucleocapsid production in infected cells and thus served as a valid marker for viral infection.

*The potency of virion-targeted IFN-β*: The antiviral potency of virus-absorbed IFNβ-ACE2 is difficult to measure; nonetheless, upper bounds can be estimated based on theoretical considerations. As shown in Figure 3 and Figure 4, NL63 exhibited stable capture of IFNβ-ACE2 when incubated with 100 pM–1 nM IFNβ-ACE2. Binding appeared stable in that virion/IFNβ-ACE2 complexes did not dissociate during extensive washing of the virus. Because one does not know the actual number of IFNβ-ACE2 molecules bound per virion, we instead calculated upper limits based on the assumption of 100% binding efficiencies, which provided an upper limit of the IFNβ-ACE2 concentration that was stably absorbed by the virus and carried into the host cell infection assay. We estimate upper limits of three IFNβ-ACE2 molecules per Spike glycoprotein × 300 Spike glycoproteins per virion × 3000 infectious virions per 100 µL culture (MOI of 0.1 in a 30,000-cell culture), which equals an upper limit of 2.7 × 10^6^ IFNβ-ACE2 molecules per 100 µL culture or an equivalent molarity of 45 femtomolar. IFNβ-ACE2 exhibited half-maximal inhibitory values in the 10 pM–1 nM concentration range in a 229E infection system, representing IFNβ-ACE2 anti-virological potency of the IFN-β domain when IFNβ-ACE2 lacks binding to the virion (Figure 6E). Based on these considerations, the antiviral activity of virus-absorbed IFNβ-ACE2 was several orders of magnitude more potent than soluble (i.e., virus non-absorbed) IFNβ-ACE2. The caveat is that virion-arrayed IFN-β is surface-bound and not in solution per se, but nonetheless this consideration points to a limited array of IFN-β having unexpectedly high bioactivity. It is currently unknown whether immobilized IFN-β may have activities that supersede soluble IFN-β on a per molecule basis, perhaps reflecting fixed-surface immobilization and increased avidity for IFNAR, thereby providing superior IFNAR crosslinking. In addition, an immobilized IFN-β array may affect IFNAR trafficking to enhance receptor signaling [63,64,65,66,67,68].

*Conclusions*: The prospect of future viral epidemics and pandemics requires advancement of new antiviral therapies. This study provides a new antiviral platform that synergistically combines the virus-anchoring activity of a specific soluble host receptor (sACE2) and an immune modulator (IFN-β) by targeting the immune modulator to the surface of infectious virions, thereby engendering highly specific antiviral activity. Overall, this study advanced the concept of an antiviral fusion protein that assembles an IFN-β shield at the virion surface to constitute a potent antiviral therapy.

## Data Availability

The original contributions presented in this study are included in the article. Further inquiries can be directed to the corresponding author(s).
